# Circadian pattern of activation of the medical emergency team in a teaching hospital

**DOI:** 10.1186/cc3537

**Published:** 2005-04-28

**Authors:** Daryl Jones, Samantha Bates, Stephen Warrillow, Helen Opdam, Donna Goldsmith, Geoff Gutteridge, Rinaldo Bellomo

**Affiliations:** 1 Intensive Care Registrar. Department of Intensive Care and Department of Surgery (Melbourne University), Austin Hospital, Melbourne, Australia; 2 Research Nurse. Department of Intensive Care and Department of Surgery (Melbourne University), Austin Hospital, Melbourne, Australia; 3 Intensive Care Consultant. Department of Intensive Care and Department of Surgery (Melbourne University), Austin Hospital, Melbourne, Australia

## Abstract

**Introduction:**

Hospital medical emergency teams (METs) have been implemented to reduce cardiac arrests and hospital mortality. The timing and system factors associated with their activation are poorly understood. We sought to determine the circadian pattern of MET activation and to relate it to nursing and medical activities.

**Method:**

We conducted a retrospective observational study of the time of activation for 2568 incidents of MET attendance. Each attendance was allocated to one of 48 half-hour intervals over the 24-hour daily cycle. Activation was related nursing and medical activities.

**Results:**

During the study period there were 120,000 consecutive overnight medical and surgical admissions. The hourly rate of MET calls was greater during the day (47% of calls in the 10 hours between 08:00 and 18:00), but 53% of the 2568 calls occurred between 18:00 and 08:00 hours. MET calls increased in the half-hour after routine nursing observation, and in the half-hour before each nursing handover. MET service utilization was 1.25 (95% confidence interval [CI] = 1.11–1.52) times more likely in the three 1-hour periods spanning routine nursing handover (*P *= 0.001). The greatest level of half-hourly utilization was seen between 20:00 and 20:30 (odds ratio [OR] = 1.76, 95% CI = 1.25–2.48; *P *= 0.001), before the evening nursing handover. Additional peaks were seen following routine nursing observations between 14:00 and 14:30 (OR = 1.53, 95% CI = 1.07–2.17; *P *= 0.022) and after the commencement of the daily medical shift (09:00–09:30; OR = 1.43, 95% CI = 1.00–2.04; *P *= 0.049).

**Conclusion:**

Peak levels of MET service activation occur around the time of routine observations and nursing handover. Our findings raise questions about the appropriate frequency and methods of observation in at-risk hospital patients, reinforce the need for adequately trained medical staff to be available 24 hours per day, and provide useful information for allocation of resources and personnel for a MET service.

## Introduction

The medical emergency team (MET) concept is an evolving hospital system change that aims to reduce morbidity and mortality in acutely ill ward patients [1-3]. The MET is most often comprised of intensive care-based staff who are mobilized by ward-based doctors and nurses to review critically ill patients on the ward. The success of the MET system relies on the assumption that early intervention in the course of clinical deterioration improves patient outcome [4]. It would be important to gain insight into the possible processes that lead to MET calls and to understand their circadian variation in order to plan appropriate staff allocation.

We recently reported that the implementation of a MET system in our hospital resulted in a 65% relative risk reduction for in-hospital cardiac arrest over a 4-month period [4]. Analysis of the pattern of activation of the MET service in that study revealed a trend toward increased activation during the evening (*P *= 0.12). Lee and coworkers [5] reported that 36% of 522 MET calls registered over a 1-year period occurred between the hours 20:00 and 08:00. No information, however, exists on the possible relationship between routine nursing or medical activity and MET calls.

Available evidence suggests that between 69% and 82% of MET calls are initiated by a nurse [5,6]. The criteria for MET activation at our institution are based on derangements in vital signs that are typically measured or assessed at times of routine nursing observation and handover. Thus, we hypothesized that activation of the MET service at our institution would cluster around these times. To test this hypothesis we analyzed the frequency of MET activation at half-hourly intervals over a 24-hour period and related this to aspects of nursing and medical daily routine.

## Materials and methods

### The hospital

Austin Health is a university-affiliated teaching hospital with three hospital campuses situated in Melbourne, Australia. The Austin Hospital is the acute care hospital in which the MET service operates. It has 400 beds and receives approximately 60,000 day and overnight admissions per year.

### Hospital emergency response teams

The acute care hospital has two levels of medical emergency responses and teams. A traditional cardiac arrest ('code blue') team is comprised of a cardiology fellow and coronary care nurse, as well as an intensive care fellow and nurse, and the receiving medical unit fellow. All wards are equipped with resuscitation trolleys containing resuscitation drugs and defibrillators.

In September 2000 a MET system was introduced into the acute campus following an extensive preparation and education process [4]. The team consists of an intensive care fellow and nurse, as well as the receiving medical unit fellow. It can be activated by any member of the hospital staff according to preset criteria for physiological instability. All code blue and MET calls are communicated by the switchboard operators through the hospital loudspeakers and paging system, and a detailed log of all calls is maintained.

### Criteria for medical emergency team activation

Calling criteria for our MET service are based on acute changes in heart rate (<40 or >130 beats/min), systolic blood pressure (<90 mmHg), respiratory rate (<8 or >30 breaths/min), conscious state, urine output (<50 ml over 4 hours), and oxygen saturation derived from pulse oximetry (<90%, despite oxygen administration). In addition, the calling criteria contain a 'staff member is worried' category to allow staff to summon senior assistance to manage any possible emergency situation.

### Outcome measures

Information on the activation of all MET calls is maintained on a hospital switchboard logbook that includes the date and time of the call, as well as the ward where the MET review occurred. The details of 2568 MET calls were manually entered into an MS Excel™ spreadsheet by two investigators who worked together and cross-checked the entries to minimize errors.

Each call was allocated to one of 48 half-hourly intervals over a 24-hour period (24:00–00:30, 00:31–01:00, 01:01–01:30, 01:31–02:00, etc.). A graph was then constructed from the 2568 episodes of MET service review to illustrate the frequency of activation at various times over the 24-hour period.

Episodes of activation were related to the periods of routine nursing handover (07:00, 13:00 and 21:00), routine nursing observations (02:00, 06:00, 10:00, 14:00, 18:00 and 22:00), and commencement and completion of the daily medical shift (08:00–18:00).

### Statistical analysis

The frequency of MET service activation during peak periods was compared with the average activation over the 24-hour period. In the case of nursing handover, the 1-hour period spanning handover (the half-hour before and the half-hour after, repeated three times per day for a total of 3 hours) was compared with the average activation over the 24-hour period. Statistical significance was determined by analysis with Fisher's exact test using MS Windows Statview (Abacus Concepts, Berkeley, CA, USA). *P *< 0.05 was considered statistically significant.

## Results

**Figure 1:**
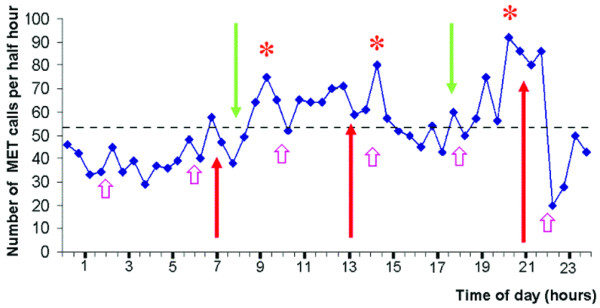
Medical emergency team (MET) calls over 24 hours. Shown is a graph illustrating the number of MET calls made per half-hour over a 24-hour period for 2568 episodes of MET review in relation to aspects of daily nursing and medical routine. Arrows demonstrate periods of nursing handover (red, up-pointing arrows), the beginning and end of the daily medical shift (green, down-pointing arrows), and periods of routine nursing observations (pink, shorter, up-pointing arrows). The dotted line represents the average number of MET calls made per half-hour interval. *P < 0.05.

During the study period (August 2000 to September 2004) there were 120,000 consecutive overnight medical and surgical admissions to the Austin Hospital and 2568 activations of the MET service. Activation of the MET service was not uniform over the 24-hour period (Fig. 1).

Over the study period, 53% of the 2568 calls occurred in the 14 hours between 18:00 and 08:00 (58% of the day). On an hourly basis, MET call utilization was more common during the hours covered by the parent unit doctors (47% of MET calls during 42% of the day). In the 5 years that the MET system has operated, there has been a trend for an increasing proportion of calls to occur after hours (18:00–08:00; Fig. 2). Thus, in 2004, 374 out of 669 (55.9%) MET calls occurred after hours, compared with 69 out of 139 (49.6%) during the year 2000 (odds ratio [OR] = 1.13, 95% CI = 0.82–1.54; *P *= 0.19).

On average there were 106 calls (2568/24) for each hour period, or 53 calls (2568/48) per half-hour period. Increased activity of the MET service was typically seen in the half-hour following routine observations, and in the half-hour before routine nursing handover (Fig. 1). A total of 401 calls were made in the three 1-hour periods spanning nursing handover. During these periods, activation of the MET service was 1.25 times more likely (95% CI = 1.11–1.52) when compared with the average activation over the 24-hour period (*P *= 0.001).

The highest level of MET service activation for any given half-hour period was seen between 20:00 and 20:30, when use of the MET service was 1.8 (95% CI = 1.25–2.48) times greater than average half-hourly utilization (*P *= 0.001). Additional peaks of activity were seen between 14:00 and 14:30 (OR = 1.53, 95% CI = 1.07–2.17; *P *= 0.022) and between 09:00 and 09:30 (OR = 1.43, 95% CI = 1.00–2.04; *P *= 0.049). All other peaks of activity failed to achieve statistical significance.

## Discussion

We report, for the first time, a detailed analysis of the level of utilization of a MET service over a 24-hour period and found a significant increase in the number of MET calls around periods of nursing handover and routine nursing observation. In addition, although MET calls occurred more frequently during the hours 08:00–18:00 (47% of calls during 42% of the day), a substantial proportion of MET calls occur after normal working hours (53% of calls during 58% of the day), with the peak time of activity occurring between 20:00 and 20:30. These findings have important implications for the frequency and method of patient monitoring, as well as for allocation of critical care resources and MET personnel, and require detailed discussion.

In a previous study at our institution [6] there was a trend toward more frequent activation of the MET service in the evening. In a study of 522 MET calls over a 1-year period, Lee and coworkers [5] demonstrated that 36% of MET calls were registered during the nightshift (20:00–8:00). Although the rate of MET calls did not vary during periods of reduced staffing, the investigators emphasized the importance of providing appropriately trained medical staff on a 24-hour basis.

In the present study, 53% of all calls occurred 'out of hours' (18:00–08:00) when wards are not staffed by parent unit doctors. In addition, there was a trend toward increased frequency of activation of the MET service during these hours in the 5 years following the introduction of the MET system. When directly compared with the study conducted by Lee and coworkers [5], 46.2% of the 2568 MET calls registered in the present study occurred between 20:00 and 08:00 hours. Our findings suggest a greater utilization of the MET service in the hours not covered by the parent unit medical staff than has previously been reported.

The frequent use of the MET service after 18:00 has important implications for allocation of resources to the MET service out of hours, and further reinforces previously reported opinion [5] that appropriately trained medical staff should be available on a 24-hour basis to assess and treat acutely ill hospital patients.

Utilization of a MET system has been associated with a reduction in all-cause hospital mortality in our institution [4,6]. Thus, our observation that MET service activation clusters around times of nursing handover and routine nursing observations raises questions about the appropriate frequency and methods of observations in 'at-risk' hospital patients. A more frequent or automated (e.g. telemetry) observation system for such at-risk patients may result in further reductions in mortality and morbidity. It is unlikely that patients would develop acute illness more frequently at specific times that happen to coincide with nursing observations or handover. It is more likely that the patient was discovered to be unwell only during a 'scheduled visit' by his/her care givers. In the case of medical staff, this would correspond to the morning medical ward round. In the case of nursing staff, we have clearly demonstrated increased levels of MET activity during periods when nurses are more likely to be tending to the patient. It is likely, therefore, that a substantial proportion of these patients would have been ill for some time before the call was made, and were only identified during routine observations or at the time of nursing handover. It is also possible that the diurnal variation of identifying 'patients in crisis' observed in the present study would not be seen in an environment with more automated and/or continuous monitoring.

The present study has a number of limitations. First, it is an observational study and does not demonstrate the effect of MET service utilization on patient outcome. However, we know from previous studies [4,6] that the introduction of the MET service was associated with significant beneficial effects on morbidity and mortality. Second, the pattern of fluctuation of the MET service at our institution is likely to be based on the calling criteria that we have implemented. The study may not apply to other hospitals where alternative calling criteria are employed. However, we deliberately employed simple calling criteria to increase the ease of utilisation of the MET system at our institution. Furthermore, the timing and frequency of patient observations reported in the study would be typical of most hospitals. Finally, information on episodes of MET review was obtained from the hospital switchboard log and did not provide information on the member of staff who activated the system. It would be interesting to known whether there was variation in the nature of the member (doctor versus nurse) and seniority of staff at various times of the day. We are currently collecting information on this aspect of MET operation.

## Conclusion

In our institution, peak levels of MET service utilization occur around the time of routine nursing observations and nursing handover, and the majority of calls occur after hours. Our findings raise questions about the appropriate frequency and technology of observations in hospital ward patients. They also provide useful information to guide appropriate resource allocation for the provision of the MET service.

## Key messages

• 	More than half of MET calls occur after hours.

• 	The peak time of MET activation is at 20:00, just before nursing handover.

• 	Other peak activities occur around nursing handover times or medical ward round times.

• 	These findings suggest that critical illness detection in hospital is episodic.

• 	More systematic approaches to hospital patient monitoring may be desirable in order to provide more timely intervention.

## Abbreviations

CI = confidence interval; MET = medical emergency team; OR = odds ratio.

## Competing interests

The author(s) declare that they have no competing interests.

## Authors' contributions

DJ conceived the study, constructed the data base, and was the principle author of the manuscript. SB, DG, and SW assisted with construction of the data base. HO, GG, and RB contributed with the study design and authorship of the manuscript. All authors read and approved the final manuscript.

## Figures and Tables

**Figure 2:**
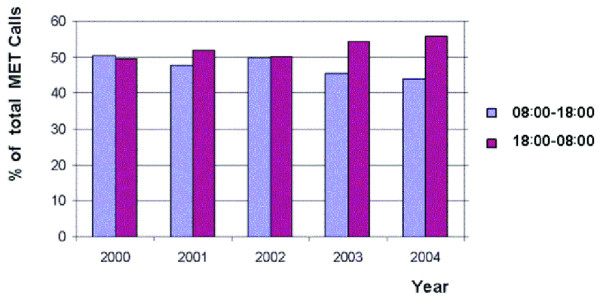
Medical emergency team (MET) calls during periods 08:00–18:00 and 18:00–08:00 comparison. Shown is a comparison of the percentage of MET calls made during the periods 08:00–18:00 and 18:00–08:00 for the years 2000–2004.
